# The Expression of the *Zonula Adhaerens* Protein PLEKHA7 Is Strongly Decreased in High Grade Ductal and Lobular Breast Carcinomas

**DOI:** 10.1371/journal.pone.0135442

**Published:** 2015-08-13

**Authors:** Jean-Christophe Tille, Liza Ho, Jimit Shah, Olivia Seyde, Thomas A. McKee, Sandra Citi

**Affiliations:** 1 Division of Clinical Pathology, Geneva University Hospitals, Geneva, Switzerland; 2 Department of Cell Biology, University of Geneva, Geneva, Switzerland; 3 Institute of Genomics and Genetics of Geneva (iGE3), University of Geneva, Geneva, Switzerland; University of Toronto, CANADA

## Abstract

PLEKHA7 is a junctional protein, which participates in a complex that stabilizes E-cadherin at the *zonula adhaerens*. Since E-cadherin is involved in epithelial morphogenesis, signaling, and tumor progression, we explored PLEKHA7 expression in cancer. PLEKHA7 expression was assessed in invasive ductal and lobular carcinomas of the breast by immunohistochemistry, immunofluorescence and quantitative RT-PCR. PLEKHA7 was detected at epithelial junctions of normal mammary ducts and lobules, and of tubular and micropapillary structures within G1 and G2 ductal carcinomas. At these junctions, the localization of PLEKHA7 was along the circumferential belt (*zonula adhaerens*), and only partially overlapping with that of E-cadherin, p120ctn and ZO-1, as shown previously in rodent tissues. PLEKHA7 immunolabeling was strongly decreased in G3 ductal carcinomas and undetectable in lobular carcinomas. PLEKHA7 mRNA was detected in both ductal and lobular carcinomas, with no observed correlation between mRNA levels and tumor type or grade. In summary, PLEKHA7 is a junctional marker of epithelial cells within tubular structures both in normal breast tissue and ductal carcinomas, and since PLEKHA7 protein but not mRNA expression is strongly decreased or lost in high grade ductal carcinomas and in lobular carcinomas, loss of PLEKHA7 is a newly characterized feature of these carcinomas.

## Introduction

Breast carcinoma is the most common cancer in women both in the developed and non-developed world, and the second leading cause of cancer death in women, after lung cancer. Breast carcinomas are classified into invasive and non-invasive, based on their infiltrating characteristics, and comprise a range of tumor types, among which ductal carcinomas are the most common, and lobular carcinomas represent about 5–10% of the total. Distinctive morphology and infiltrative pattern between ductal and lobular carcinomas are useful to make the diagnosis. Furthermore, E-cadherin and p120ctn immunohistochemistry can be used to distinguish ductal from lobular carcinomas, since the vast majority of lobular carcinomas fail to express E-cadherin, while p120ctn shows a cytoplasmic distribution [1–3]. Recent studies confirm that the molecular hallmark of lobular carcinoma compared to ductal carcinoma is the loss of function of E-cadherin, associated with changes in the expression of genes controlling cytoskeleton remodeling, cell adhesion and extra cellular matrix-interaction pathways [4, 5]. Indeed, loss of cell-cell adhesion, as well as enhanced migration and remodeling of cell and tissue architecture, is of fundamental importance in the local invasiveness and metastatic spread of cancer cells [6]. In epithelial tissues cell-cell adhesion relies primarily on *zonulae adhaerentes* (ZA) and desmosomes, which contain cadherin family adhesion molecules, and are associated with apical tight junctions (TJ, or *zonulae occludentes*) at the epithelial junctional complex [7].

E-cadherin is the major transmembrane molecule of *zonulae adhaerentes*, and interacts in the cytoplasm with a protein machinery, which connects it to the actin and microtubule cytoskeletons [8]. The expression of E-cadherin is controlled at the transcriptional and post-transcriptional levels. For example, loss of function of the E-cadherin gene in breast lobular carcinomas has been reported to be due to gene promoter methylation, mutation, and allelic loss [9]. Another mechanism of regulation of E-cadherin expression and function is the control of its retention at the cell surface. The cytoplasmic protein p120ctn binds to the E-cadherin juxta-membrane domain, thus preventing E-cadherin endocytosis, and stabilizing it at the cell surface. Consistently, the loss, mislocalization, aberrant activity or specific isoform expression of p120ctn may be linked to poor tumor prognosis, through the modulation of E-cadherin internalization [10–12].

E-cadherin was recently shown to be stabilized at epithelial junctions by a protein complex that links it to the microtubule cytoskeleton, and which includes p120ctn, paracingulin, and the recently discovered protein PLEKHA7 [13–15]. PLEKHA7 interacts not only with p120ctn and paracingulin, but also with the adherens junction protein afadin [16] and the microtubule minus-end capping protein nezha [13]. In addition, it forms a complex with the TJ proteins cingulin and ZO-1 [17]. Importantly, unlike E-cadherin and associated catenins (p120ctn, α-catenin and β-catenin), but similarly to paracingulin and afadin, PLEKHA7 is not distributed along lateral contacts (*puncta adhaerentia*) of polarized epithelial cells, but exclusively at circumferential apical *zonulae adhaerentes* (ZA) [14]. Indeed, the “zonular”, belt-like distribution of PLEKHA7 is remarkably similar to, but not completely overlapping with, the localization of the TJ markers ZO-1 and cingulin [14]. This observation suggests that stabilization of E-cadherin by the microtubule cytoskeleton occurs only at the ZA, in a specific molecular environment that may also require neighbouring TJ proteins. Of note, genetic studies have implicated PLEKHA7 in hypertension [18, 19] and primary angle closure glaucoma [20]. Moreover, depletion studies in zebrafish embryos indicate a role of PLEKHA7 in cardiac morphogenesis and contractility [21]. Therefore, PLEKHA7 appears to be involved not only in the stabilization of adhesive protein complexes, but also in important physiological and pathological processes. However, the molecular mechanisms implicating PLEKHA7 in organ physiology and pathology are not clear.

Since PLEKHA7 is implicated in the stabilization of E-cadherin complex, and the expression and activities of both E-cadherin and the PLEKHA7-interacting protein p120ctn are linked to tumor formation and metastasis [22], we sought to explore if and how PLEKHA7 expression is altered in epithelial cancer. To this purpose, here we studied the expression and localization of PLEKHA7 in human breast invasive ductal and lobular carcinomas by immunohistochemistry, immunofluorescence, and quantitative RT-PCR. Our results show that PLEKHA7 protein expression and accumulation at sites of cell-cell contact confirm its zonular localization in human normal and cancer epithelial cells, decreases with increasing grade in ductal breast carcinomas, and is lost in lobular carcinomas, in a pattern that distinguishes it from other markers of both ZA and TJ.

## Materials and Methods

### Tissue samples

Formalin fixed paraffin embedded (FFPE) and frozen tissue samples of normal and breast carcinoma tissues were obtained from the archives of the Division of Clinical Pathology at the Geneva University Hospital, according to a protocol approved by the Geneva Cantonal Committee for Research Ethics (CCER) (Protocol number 14–272, Project title “Expression of junctional proteins in breast cancer”), and which has no requirement for written informed consent, because it is a retrospective study. Patient records and information was anonymized and de-identified prior to analysis.

Tumor type was assessed according to the World Health Organization criteria [23], grade according to the Nottingham criteria [24], estrogen and progesterone receptor expression was reported with the Allred scoring system, and considered positive if Allred score ≥3 [25], and HER2/neu status (HER2/neu considered amplified if by fluorescence in-situ hybridization a HER2/Cep17 ratio > 2.0).

### Antibodies

The following antibodies were used for immunocytochemistry and/or immunofluorescence: mouse anti-PLEKHA7 (monoclonal 37-8F1 culture supernatant, undiluted for IHC), rabbit anti-PLEKHA7 (20943, 1:250, only for immunofluorescence), rabbit anti-cingulin (R39 or C532, 1:500), mouse anti-cingulin (Invitrogen 37–4300, 1:100), mouse anti-p120-ctn (15D2, a kind gift of Prof. Al Reynolds, Vanderbilt University, USA, 1:250) [14, 26–28], mouse anti E-cadherin (BD 610182) and mouse anti-ZO-1 (BD610966) [14]. Alexa-488 and Cy3 labelled secondary antibodies for immunofluorescence were from Jackson Immunoresearch Laboratories Europe (www.JIREurope.com).

### Cell culture and transfection with siRNA

Culture of the human colon carcinoma cell line SKCO15 (a generous gift from A. Nusrat, Emory University, cells originally from American Type Culture Collection, Manassas, VA) was carried out as described previously [29]. Transient depletion of PLEKHA7 using siRNA was carried out by transfection with Lipofectamine RNAiMax (Invitrogen). Three distinct siRNAs against PLEKHA7 were used, which gave the same results by immunofluorescence, using the 37-8F1 antibody. The oligos targeted the following sequences of human PLEKHA7: 1) 5’- GGCATGAGGACCTACTACT-3’; 2) 5’-CCTACTACTTCAGTGCCGA-3’; 3) 5’-CCTACCTCCAGCTGAAGAA-3’. siRNA control was obtained from Sigma (SIC001). Preparation of cell lysates and immunoblotting to assess PLEKHA7 expression in normal and transfected cells was carried out as described previously [29, 30].

### Immunohistochemistry and immunofluorescence

Immunohistochemical staining of 5 μm-thick sections of formol-fixed, paraffin-embedded samples was carried out using the Ventana BenchMark ULTRA Automated IHC/ISH Slide Staining Module (Roche), according to the U ultraView DAB procedure. The antigen retrieval protocol was optimized for each antigen: for PLEKHA7 heat-treatment (64 min) was followed by treatment with the endopeptidase Protease 3 (Roche 760–2020), whereas only heat treatment, for either 64 min or 52 min, was used for E-cadherin and ZO-1, respectively. Sections were counter-stained with hematoxylinII and post counter-stained with bluing reagent. PLEKHA7 staining was considered a) positive, when it bordered the lumen continuously; b) reduced, when interrupted, focal areas of labeling were observed, and c) negative, when no luminal or junctional labeling, either continuous or interrupted, was observed.

For immunofluorescence of tissue sections, 5 μm-thick frozen sections (stored at -80°) were thawed, air-dried, fixed with acetone at -20°C for 20 min, rehydrated in PBS, incubated with primary and secondary antibody (incubations 1 hr at 30°C, each followed by three washes in PBS), and mounted in Pro-Long anti-fade medium (Invitrogen) containing 1.5 μg/ml DAPI [14].

For immunofluorescence on cultured cells, confluent monolayers grown on round glass coverslips were permeabilized and fixed with cold methanol (-20°C) for 10 min, followed by rehydration in PBS, incubation with antibodies as above, and mounting in Vectashield mounting medium. Specimens were analyzed with a Zeiss LSM700 confocal microscope, in multi-tracking mode [14].

### Real time RT-PCR

Frozen samples of ductal and lobular breast carcinoma were analyzed histologically by hemalum and eosin staining to identify relevant areas. Total RNA was extracted from 5–10 20 μm-thick sections of each sample, using the miRNeasy Mini Kit (Qiagen). cDNA was synthesized using 300 ng of total RNA, as measured by Nanodrop ND-1000 (NanoDrop Technologies, Inc., USA) with 12.5 pM of oligo-dT primers and 25 pM of random 6 mers, in a total reaction volume of 10 μl, following the manufacturer’s protocol (TAKARA Bio. Inc.). Quantitative PCR using 0.25 μl of cDNA for each reaction was performed in triplicates using SYBR Green reagents and a 3-step PCR program: 45 cycles of 95°C, 10s; 60°C, 10s; 72°C, 10s, on a Light-Cycler 96 (ROCHE). The references genes for the expression of CDH1 and PLEKHA7 were chosen with the help of the database Genevestigator (https://www.genevestigator.com/gv/). Primer pairs with efficiency >1.95 were selected: PLEKHA7 forward 5’- GACGGGACAGTTTTCTCCAG -3’, reverse 5’- TTGGTTTCTGTCTTGCTTCG-3’; E-cadherin (CDH1) forward 5’- CAGCACGTACACAGCCCTAA -3’, reverse 5’- TGTCCCTGTTCCAGTAGCAA -3’; FAM188B forward 5’- GACTTTGATGTCCCCACCAG -3’, reverse 5’- TTCCCAGTCAGGAGCAGATT -3’. Values were analyzed using Relative Analysis Quantification software, and expressed as ratios of relative concentration.

### Statistical analysis

A p-value of <0.05 was considered as significant. Z-score, Mann-Whitney U-Test and t-test were performed on the The Social Science Statistics web site (http://www.socscistatistics.com/Default.aspx).

## Results

**Fig 1:**
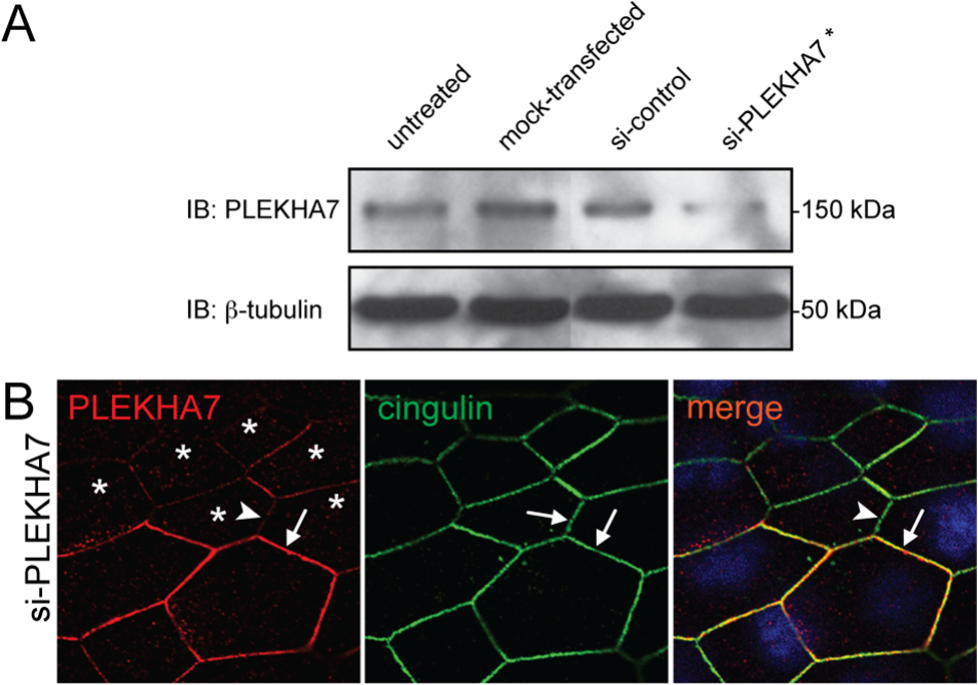
Specificity of anti-PLEKHA7 monoclonal antibody for human PLEKHA7. (A) Immunoblot analysis of lysates from cultured human epithelial cells (SKCO), following treatment with siRNA for PLEKHA7 (siRNA3), control siRNA (untreated and mock-transfected cells for control) using monoclonal antibody 37-8F1. (B) Immunofluorescence analysis of si-PLEKHA7 treated cultures, showing decreased PLEKHA7 junctional (red, Cy3) labeling in PLEKHA7-depleted cells (asterisks, siRNA2) with respect to normal cells. Anti-cingulin antibody (green, Alexa488) was used as control to normalize junctional labeling. The same results were used with cells depleted of PLEKHA7 by three different siRNAs (see Materials and Methods).

To study PLEKHA7 expression in human breast carcinomas, we examined cohorts of non lobular, predominantly ductal carcinoma, and lobular breast carcinomas of different stage, grade and hormonal status (Table 1). To examine the expression and localization of PLEKHA7 by immunohistochemistry and immunofluorescence, we used a monoclonal antibody against human PLEKHA7, which was previously used for experiments on rodent cells and tissues [14]. To validate the specificity of this antibody for human tissues, we examined its reactivity using cultured human epithelial cells depleted of PLEKHA7 through siRNA expression. Immunoblot analysis showed a decreased intensity of the PLEKHA7 polypeptide in lysates of cells depleted of PLEKHA7, whereas cells treated with control-siRNA or mock-transfected showed no reduction in the levels of PLEKHA7, when compared to untreated cells (Fig 1A). Next, we labeled by double immunofluorescence, using anti-PLEKHA7 and anti-cingulin antibodies (as control to normalize for zonular junctions), cultures of cells treated with anti-PLEKHA7 siRNA. Here, we could detect PLEKHA7-depleted cells (asterisks in Fig 1B) showing decreased junctional labeling for PLEKHA7 (arrowhead in Fig 1B) adjacent to neighbouring cells, where the cingulin labeling showed the same intensity as that in PLEKH7-depleted cells, whereas the PLEKHA7 labeling was stronger (arrow in Fig 1B), indicating that in these latter cells PLEKHA7 expression was not silenced. The decreased PLEKHA7 labeling, in the presence of similar intensity of cingulin labeling, indicated a bona fide reduction of junctional PLEKHA7, rather than an overall reduction of junctional protein accumulation. Taken together, the immunoblotting and immunofluorescence experiments using cultures depleted of PLEKHA7 by siRNA demonstrated that the monoclonal antibody 37-8F1 is specific for human PLEKHA7.

**Table 1:** Classification of breast carcinomas used in this study.

		Non-ILC (n = 121)	ILC (n = 125)
Tumor type	IDC	113	
	microapillary	3	
	mixte IDC and micropapillary	4	
	mixte IDC and mucinous	1	
Tumor grade	G1	41	0
	G2	42	119
	G3	38	6
Hormonal status	ER+/PR+	84	102
	ER+/PR-	10	14
	ER-/PR+	2	0
	ER-/PR-	24	2
	ND	1	7
Ki67	<14%	60	81
	>14%	60	39
	ND	1	7
HER2 amplification	Positive	20	13
	Negative	97	105
	ND	4	7

IDC = ductal carcinomas; ILC = lobular carcinomas

**Fig 2:**
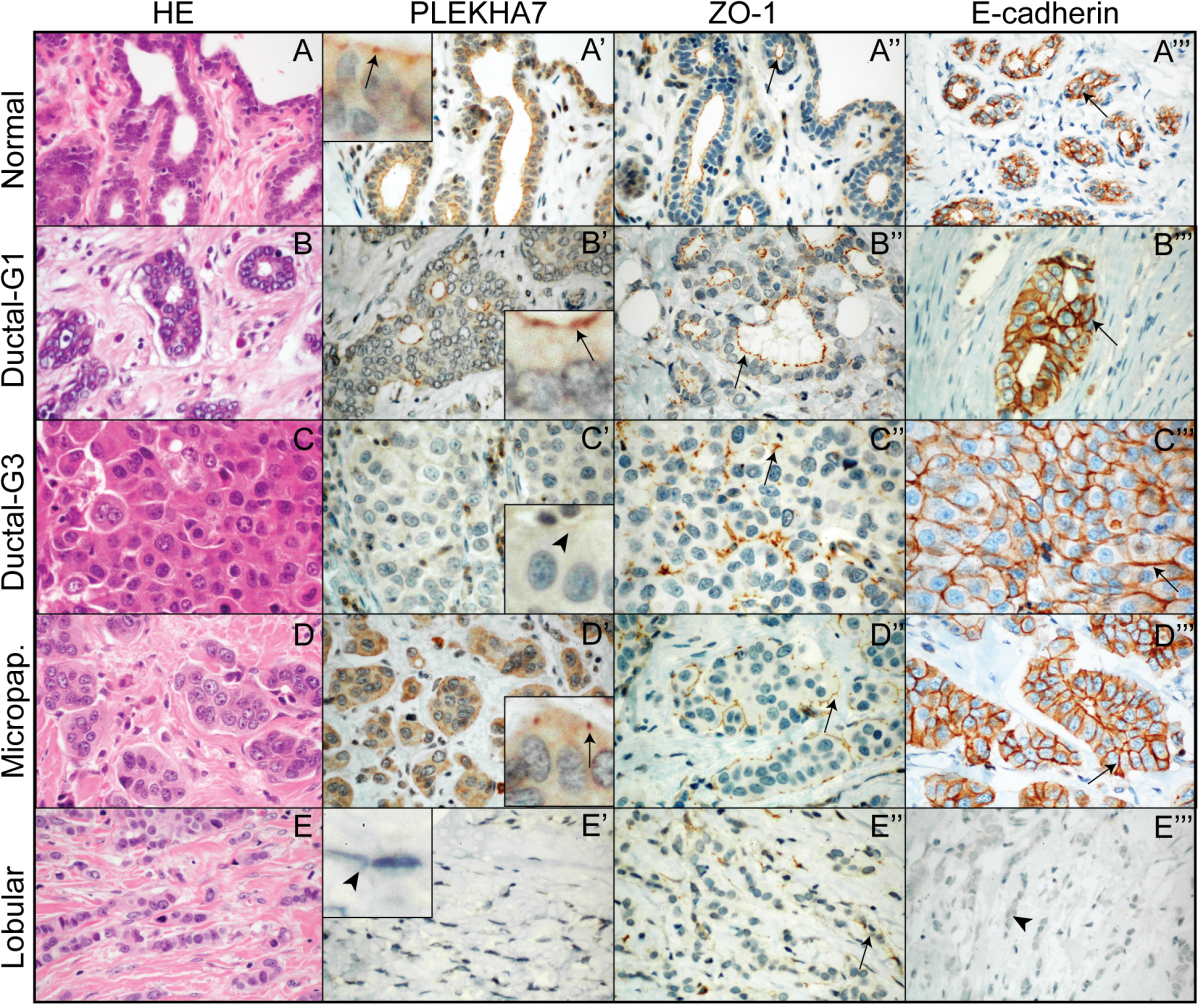
Immunohistochemical localization of PLEKHA7 in human breast carcinomas. Images show sections of normal breast tissue (normal, A-A”‘), ductal carcinoma of low (G1, B-B”‘) and high (G3, C-C”‘) grade, micropapillary areas of ductal carcinoma (D-D”‘), and lobular carcinomas (E-E”‘), after HE staining (A-E), or immunohistochemical labeling with antibodies against PLEKHA7 (A’-E’), ZO-1 (A”-E”) or E-cadherin (A”‘-E”‘). Arrows in magnified insets indicate apical zonular junctional labeling for PLEKHA7. Arrowheads show weak or undetectable labeling. Sections were observed at original magnifications between 10x and 40x.

To examine PLEKHA7 expression in breast cancers, we carried out immunohistochemical localization of PLEKHA7 in paraffin sections of normal breast tissue, ductal carcinomas of increasing grade (G1, G2, G3), including those with micropapillary patterns, and lobular carcinomas, using the monoclonal antibody 37-8F1. In the same tissues we studied the distribution of ZO-1, a marker of zonular apical TJ, and E-cadherin, a marker of both apical ZAs, and of lateral contacts of epithelial cells [14] (S1 Table). In normal breast tissue, PLEKHA7 was detected at apical zonular junctions of epithelial cells facing the lumen of glands (Fig 2A’, arrow in magnified inset). A similar zonular localization was detected for ZO-1 (arrow in Fig 2A”), whereas E-cadherin was localized not only at zonular junctions, but also also along the lateral contacts of epithelial cells (arrow Fig 2A”‘). The localization of PLEKHA7, ZO-1 and E-cadherin in the glandular structures of well-differentiated ductal carcinomas was very similar to that of normal breast tissue, for example PLEKHA7 was accumulated at the apical zonular belts of the cells lining the lumen of the tubules (Fig 2B’–2B”‘, arrows). In contrast, in poorly differentiated (G3) ductal carcinomas the expression of PLEKHA7 was absent or hardly detectable in the apical area of cells, which faced apparently aborted lumens (Fig 2C’, arrowhead in inset). The loss or reduced expression of PLEKA7 in high grade (G3) ductal carcinoma compared to well differentiated (G1) ductal carcinoma was statistically significant (p = 0.0028). In these structures, ZO-1 expression was detected apically (arrow in Fig 2C”), whereas E-cadherin expression was detected throughout all the area of cell-cell contact (arrow in Fig 2C”‘). In tumors showing an invasive micropapillary pattern, the reversal of the apicobasal polarity [31] was underlined by the localization of zonular junctional PLEKHA7, as well as ZO-1, on the sides of epithelial cells facing the stroma (arrows Fig 2D’–2D”), instead of the interior of cell clusters, even when an internal tubular structure was visible. In contrast, E-cadherin was still distributed along all the lateral contacts (arrow in Fig 2D”‘). Strikingly, no PLEKHA7 expression was detected in lobular carcinomas (Fig 2E’, arrowhead in inset). Typically no E-cadherin labeling was detected in lobular carcinomas (arrowhead in Fig 2E”‘), however even in the rare cases (n = 9, 9.6%) where E-cadherin was expressed, no PLEKHA7 labeling was detected (S1 Table). ZO-1 expression was very low, and barely detectable in lobular carcinomas, in dot-like or short linear patterns (arrow in Fig 2E”). No correlation was observed between PLEKHA7 expression and ER status (Fisher exact test statistic value = 0.58), PR status (value = 1), or HER2 status (value = 0.58).

**Fig 3:**
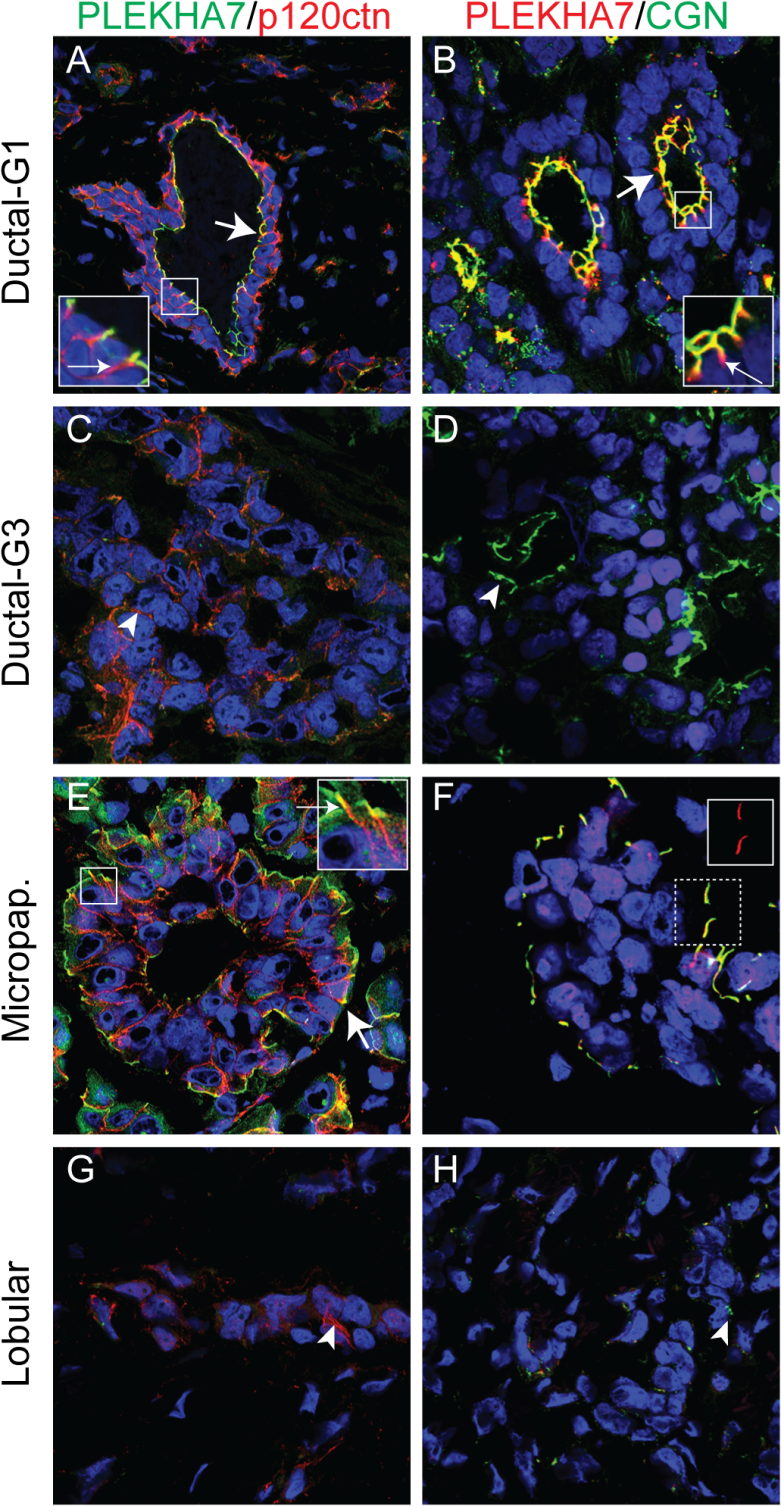
Immunofluorescent localization of PLEKHA7 in human breast carcinomas. Double immunofluorescent localization of either PLEKHA7 (green, Alexa488) and p120-catenin (p120ctn) (red, Cy3) (A, C, E, G), or PLEKHA7 (red, Cy3) and cingulin (green, Alexa488) (B, D, F, H) in frozen sections of human breast carcinomas. Arrows (see also magnified insets) indicate sites of partially overlapping localization of PLEKHA7 with either p120ctn or cingulin at apical junctions. Arrowheads indicate sites devoid of PLEKHA7 labeling, but still showing detectable p120ctn/cingulin labeling. The inset in panel F shows individual PLEKHA7 labeling in an area where it is co-localized with cingulin. The arrowhead in G shows cytoplasmic p120ctn labeling. Nuclei are labeled by DAPI in blue. Original magnification 40x.

Next, to explore an alternative method of immunochemical localization, and analyze a different set of junctional markers, we labeled frozen sections of breast carcinomas by double immunofluorescence. Sections were labeled either with antibodies against PLEKHA7 and the adherens junction marker p120ctn, or with antibodies against PLEKHA7 and the TJ marker cingulin (Fig 3) [26, 32]. In ductal carcinomas, p120ctn and PLEKHA7 localization overlapped at the ZA (arrow and magnified inset in Fig 3A), however, as previously shown in rodent epithelia [14] p120ctn was also localized, similarly to E-cadherin, to lateral contacts (red labeling indicated by small arrow in Fig 3A, inset). In contrast, cingulin was partially co-localized with PLEKHA7 at zonular apical junctions (arrow and magnified inset in Fig 3B), but was absent from lateral contacts, whereas PLEKHA7 labeling was also detected immediately below the TJ, in the absence of cingulin (red labeling indicated by small arrow in Fig 3B, inset). In high grade (G3) ductal carcinomas PLEKHA7 labeling was low or undetectable, whereas both p120ctn and cingulin could be detected at the areas of contact between epithelial cells, albeit in non-overlapping patterns (arrowheads in Fig 3C and 3D). Confirming the results on paraffin sections, PLEKHA7- and cingulin-containing junctions were detected on the reversed polarity cells of micropapillary carcinomas, and only p120ctn was detected alongside lateral contacts, similarly to E-cadherin (arrows and magnified insets in Fig 3E and 3F). Again supporting the immunohistochemistry results, PLEKHA7 labeling was undetectable in frozen sections of lobular carcinoma (Fig 3G and 3H). In contrast, p120ctn labeling in lobular carcinomas was detected, albeit weak and mainly cytoplasmic, with no apparent junctional localization, whereas cingulin labeling was detectable in a dot-like or short segment pattern (arrowheads in Fig 3G and 3H). In summary, the immunofluorescence analysis confirmed that i) PLEKHA7 expression was restricted to the apical region of epithelial gland cells, either facing the lumen in normal ductal carcinomas, or facing the stroma in the inverted polarity micropapillary carcinomas, ii) was distinct from that of either E-cadherin/p120ctn, or cingulin/ZO-1, and iii) was undetectable in lobular carcinomas.

**Fig 4:**
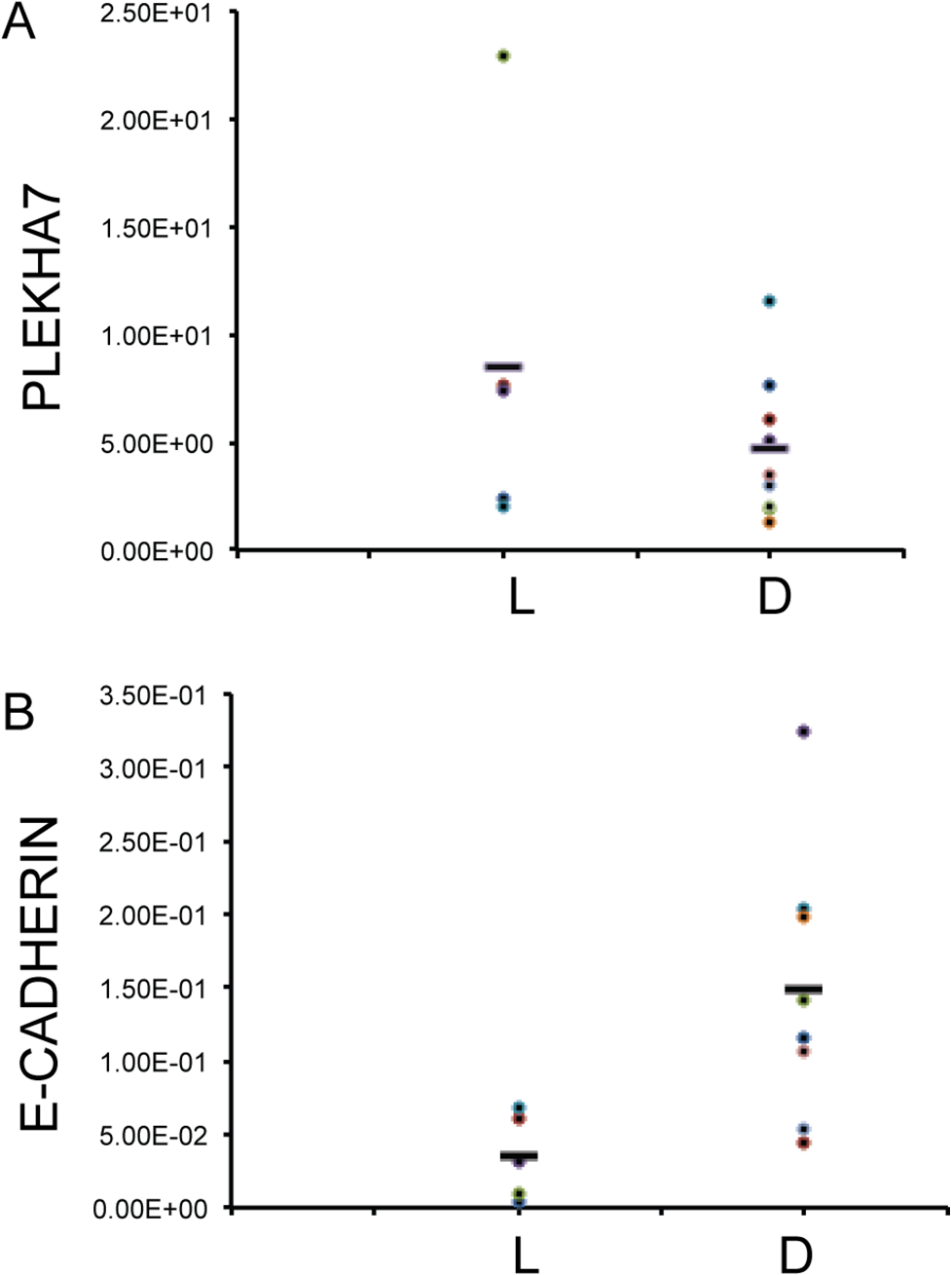
PLEKHA7 transcript quantification. Plots showing relative mRNA concentrations for PLEKHA7 (A) and E-cadherin (B), in lobular (L) versus ductal (D) breast carcinomas, taking the gene FAM188 as an internal reference.

Finally, we sought to explore whether the decreased or undetectable PLEKHA7 protein expression in specific carcinomas could be due to reduced or undetectable mRNA levels, by carrying out quantitative RT-PCR on frozen tumor samples. E-cadherin mRNA levels were also measured, and expression levels were normalized to the expression of internal, reference genes with the same range of expression in breast tissues. PLEKHA7 mRNA was detected in both ductal and lobular carcinomas in similar amounts, and levels of PLEKHA7 mRNA did not show a significant correlation with tumor grade (Fig 4). In contrast, E-cadherin mRNA levels were statistically significant (p = 0.034) and consistently lower in lobular carcinomas, when compared to ductal carcinomas, as expected (Fig 4). This indicated that the decreased or undetectable expression of PLEKHA7 in high grade ductal carcinomas and lobular carcinomas is not due to decreased mRNA levels.

## Discussion

PLEKHA7 is a recently characterized component of the *zonula adhaerens*, and its involvement in different pathological processes make it an extremely interesting protein to study, to clarify the mechanistic involvement of the protein machinery of cell-cell junctions, and more specifically of the “zonular signalosome” [33] in disease. This is an important field of investigation, as indicated by the growing interest in the expression of adherens and tight junction proteins in cancer [34–36]. Here we aimed to characterize the expression and localization of PLEKHA7 in human breast carcinomas, based on the notions that i) PLEKHA7 participates in the stabilization of the E-cadherin-associated zonular junctional protein complex, and ii) E-cadherin plays a fundamental role in the formation and metastatic spread of epithelial tumors. Our results, showing that PLEKHA7 accumulates at junctions of epithelial cells forming gland or tubular structure, indicate that PLEKHA7 expression can be used to more precisely grade tubular formation in ductal carcinoma. In addition, since PLEKHA7 immunolabeling is strongly decreased in poorly differentiated ductal carcinoma and undetectable in lobular carcinoma, where no tubules are formed, PLEKHA7 could be proposed as an additional marker, keeping in mind that lobular carcinoma is still best considered a morphological diagnosis [3].

Loss of E-cadherin expression is typical of lobular carcinomas, where the E-cadherin gene is often mutated or methylated, defining E-cadherin as a bona fide tumor suppressor of the lobular breast cancer subtype [22, 37]. We could not detect PLEKHA7 labeling in lobular carcinomas despite the occurrence of PLEKHA7 mRNA. We speculate that the loss of PLEKHA7 protein expression could be due to inhibited translation of PLEKHA7 mRNA, or increased instability of the protein. This, in turn, could be due to the altered interaction of PLEKHA7 with its binding partners at the ZA, for example p120ctn [28] and afadin [38], both of which have been implicated in tumorigenesis. Importantly, afadin and p120ctn regulate the invasive phenotypes of breast cancer cells [39] [40, 41], for example an imbalance in the expression of E-cadherin versus specific p120ctn isoforms promotes invasive dissemination and metastasis [12]. Therefore, since PLEKHA7 interacts with p120ctn [13], the loss of PLEKHA7 might contribute to tumor invasiveness, by altering the stability and signaling output of p120ctn [42].

Our immunohistochemistry and immunofluorescence observations are at variance with results reported in a previous study [43], where a commercial rabbit antiserum against PLEKHA7 did not show any labeling in normal breast tissue and low grade ductal carcinomas, but showed strong labeling in high grade ductal carcinomas and lobular carcinomas. These differences may be due to antibody specificity and/or different antigen retrieval and staining protocols. Of note, the PLEKHA7 labeling of lobular carcinomas described by [43] was cytoplasmic. In contrast, we did not observe cytoplasmic staining for PLEKHA7 above background, in either normal breast or ductal or lobular carcinoma, using both frozen and formalin fixed paraffin embedded sections. Instead, we detected PLEKHA7 labeling at the ZA of epithelial cells, in a linear or dot-like pattern, similar to other zonular proteins (ZO-1, cingulin), and in agreement with previous observations on rodent and human epithelial cells and tissues [13–15]. The consistence of our results with the established localization of PLEKHA7 in normal epithelia, together with the specificity of the monoclonal antibody used here, supports our data. Whether PLEKHA7 can exist in a diffuse cytoplasmic form either in cultured cells or tissues remains to be further investigated. However our results suggest that when PLEKHA7 is not associated with the cytoskeleton- and/or the E-cadherin-associated zonular protein complex, it becomes unstable and is degraded.

The loss of PLEKHA7 protein expression despite normal mRNA levels underlines the concept that transcript levels in normal and pathological tissues do not necessarily correlate with protein levels, which are instead likely to be more accurately quantified by proteome analysis. In this perspective, a recent proteomic analysis of human breast cancer progression in cultured cells showed that invasive cells are indeed characterized by a collapse of proteins of the adhesive machinery [44]. It is also important to consider that it is not simply the level of expression of a protein, but also its localization and activity, which may be crucial for its correct functioning in any cellular context. This may in turn be subjected to regulatory control mechanisms, for example through associations with other proteins, which may also control stability. With this in mind, we examined the relationships between the expression and localization of PLEKHA7 with that of the zonular tight junction proteins ZO-1 and cingulin, and the adherens junction proteins E-cadherin and p120ctn, with which PLEKHA7 forms complexes [13, 17]. We found that when PLEKHA7 was expressed, its subcellular localization was very similar to, although not precisely overlapping with, that of ZO-1 and cingulin, at apical *zonulae*. This confirms the exclusive association of PLEKHA7 with zonular adherens junctions, and not with lateral contacts, distinguishing it from E-cadherin and catenins [14]. The occurrence of detectable ZO-1 and cingulin labeling, but low or no PLEKHA7 labeling in poorly differentiated ductal carcinomas and in lobular carcinomas, together with the aberrant localization of ZO-1 and cingulin in these tumors, suggests that abortive TJ-like structures can form independently of the formation of a neighbouring PLEKHA7-containing ZA (see also [26, 32]). Moreover, it suggests that E-cadherin and p120ctn, when expressed in this context, may not be stabilized in a zonular structure by anchoring to the microtubule cytoskeleton, and might therefore have altered signaling activities. Proteins at zonular epithelial junctions fulfill a wealth of signaling functions, by interacting, among others, with regulators of Rho family GTPases, and transcription factors [33]. So, additional studies using different model systems will be important to examine how PLEKHA7 contributes to regulating signaling by zonular and non-zonular junctional proteins, and cell migration, invasive behavior, and other diseases.

## Supporting Information

S1 Table Summary of data. Summary of data for ductal carcinomas (IDC) and lobular carcinomas (ILC) analyzed in this study, with respect to histological type (type), tumor grade (grade), pathological size (pT) according to TNM classification 7^th^ Edition 2010 (T), pathological node (pN) according to TNM classification 7^th^ Edition 2010 (N), Allred score (ER, PR), proliferation index (Ki67) in percentage (MIB1), HER2 status (HER2), immunofluorescence performed (IF), RT-PCR performed (RT-PCR), IHC scoring (E-cadherin, PLEKHA7, ZO-1).
(XLSX)
